# Serum YKL-40 Levels Correlate with Infarct Volume, Stroke Severity, and Functional Outcome in Acute Ischemic Stroke Patients

**DOI:** 10.1371/journal.pone.0051722

**Published:** 2012-12-14

**Authors:** Hyun Young Park, Chang-Duk Jun, Se-Jeong Jeon, See-Sung Choi, Hak-Ryul Kim, Dan-Bee Choi, Seongae Kwak, Hak-Seung Lee, Jin Sung Cheong, Hong-Seob So, Young-Jin Lee, Do-Sim Park

**Affiliations:** 1 Department of Neurology, School of Medicine, Wonkwang University, Iksan, Korea; 2 School of Life Sciences, GIST, Gwangju, Korea; 3 Department of Radiology, School of Medicine, Wonkwang University, Iksan, Korea; 4 Department of Internal Medicine, School of Medicine, Wonkwang University, Iksan, Korea; 5 Department of Laboratory Medicine, School of Medicine, Wonkwang University, Iksan, Korea; 6 Department of Microbiology, School of Medicine, Wonkwang University, Iksan, Korea; 7 Center for Metabolic Function Regulation, Institute of Wonkwang Medical Science, and Institute of Wonkwang Clinical Medicine, School of Medicine, Wonkwang University, Iksan, Korea; University of Nebraska Medical Center, United States of America

## Abstract

**Background and Purpose:**

YKL-40 is associated with various neurological disorders. However, circulatory YKL-40 levels early after onset of acute ischemic stroke (AIS) have not been systematically assessed. We aimed to identify the temporal changes and clinical usefulness of measuring serum YKL-40 immediately following AIS.

**Methods:**

Serum YKL-40 and C-reactive protein (CRP) levels were monitored over time in AIS patients (n = 105) and compared with those of stroke-free controls (n = 34). Infarct volume and stroke severity (National Institutes of Health Stroke Scale; NIHSS) were measured within 48 hours of symptom onset, and functional outcome (modified Rankin Scale; mRS) was measured 3 months after AIS.

**Results:**

Within 12 hours of symptom onset, levels of YKL-40 (251 vs. 41 ng/mL) and CRP (1.50 vs. 0.96 µg/mL) were elevated in AIS patients compared to controls. The power of YKL-40 for discriminating AIS patients from controls was superior to that of CRP (area under the curve 0.84 vs. 0.64) and YKL-40 (r = 0.26, *P*<0.001) but not CRP levels were correlated with mRS. On day 2 of admission (D2), YKL-40 levels correlated with infarct volume and NIHSS. High YKL-40 levels predicted poor functional outcome (odds ratio 5.73, *P* = 0.03). YKL-40 levels peaked on D2 and declined on D3, whereas CRP levels were highest on D3.

**Conclusions:**

Our results demonstrate serial changes in serum YKL-40 levels immediately following AIS and provide the first evidence that it is a valid indicator of AIS extent and an early predictor of functional outcome.

## Introduction

YKL-40, also known as human chitinase-like protein 1 (HC-gp39), is a novel cerebrospinal fluid biomarker of various neuronal diseases, including Alzheimer’s disease, meningitis, and traumatic brain injury [1]–[5]. In addition, elevated plasma YKL-40 levels are associated with increased risk of ischemic stroke in the general population [6].

Diverse inflammatory and tissue remodeling conditions, including atherosclerosis, are associated with elevated YKL-40 expression levels in infiltrating macrophages [4]–[8]. Interleukin (IL)-1β and tumor necrosis factor (TNF)-α are produced from macrophage in neuroinflammatory conditions [4], [9], [10], and both cytokines are capable of upregulating YKL-40 expression in reactive astrocytes or macrophages [5]. Unlike the general findings for YKL-40 and macrophages, the cell type in which it is produced, in other inflamed tissues, neuroinflammatory diseases show tissue-specific features of YKL-40 and its producing cell type. Indeed, a recent study showed the abundant expression of YKL-40 by astrocytes but not by macrophages in brain tissue from patients with acute bacterial meningitis and progressive multiple leukoencephalopathy [5].

Despite the increased recognition of an association between YKL-40 and neurological disorders [1]–[6], it is not yet known how circulatory YKL-40 changes early after acute ischemic stroke (AIS) onset or whether it is a feasible biomarker for AIS in clinical practice. We aimed to identify the temporal dynamics of circulatory YKL-40 levels immediately following AIS, the relationship between YKL-40 levels and 3 key clinical parameters (infarct volume, stroke severity, and functional outcome), and the practical clinical application of YKL-40 in AIS by comparing it with C-reactive protein (CRP). CRP, a sensitive indicator of systemic inflammation, has been shown to be powerful indicator of stroke severity and functional outcome of AIS [11]–[16].

## Subjects and Methods

### Patient and Control Selection

The study protocol (Wonkwang university hospital approval number 1276) was performed in accordance with the Institutional Guidelines for Human Research and all participants provided written informed consent prior to their participation. For participants lacking mental or physical capacity to consent, a legal proxy provided written informed consent on behalf of the participant.

Cases were consecutive Korean patients (>19 years old) admitted to a single hospital with AIS within 12 hours of symptom onset. All cases belonged to 1 of the 3 major subtypes (large artery atherosclerosis or atherothrombosis [LAA], small-vessel occlusion or lacunar [SVO], or cardioembolic [CE]) of the Trial of Org 10172 in Acute Stroke Treatment criteria [17]. Exclusion criteria were: (1) no neuroimaging within 48 hours of admission; (2) 2 or more causes of stroke; (3) primary intracerebral hemorrhage, post-seizure neurologic deficit, brain abscess, or tumor; (4) recent history of infection as an outpatient, obvious signs of hospital-acquired infection, or body temperature >38.0°C on admission; (5) myocardial infarction, surgery, or trauma in the previous 30 days; (6) specified comorbidities (end-stage renal/hepatic disease, metastatic malignancy, autoimmune disorder); and (7) treatment with steroids or other immune suppressive agents during the week prior to the study.

The controls were age-comparable volunteers who attended a health screening in the health-promotion center of the same hospital. Participants were required to have no neurological deficits or neurological symptoms during the previous 3 months and no history of previous stroke, transient ischemic attack, or carotid stenosis. The same exclusion criteria, regarding the presence or a history of medical disease, specified comorbidities, and medication use, which was used for the acute ischemic stroke group, was also applied to the control group.

### Definition of *Vascular Risk Factors*

Age was categorized as <65 and *≥*65 years for logistic analysis. Hypertension was defined as systolic blood pressure ≥140 mmHg, diastolic pressure ≥90 mmHg, or current use of antihypertensive medication. Diabetes mellitus was defined as a fasting glucose level of ≥7.0 mmol/L or current use of hypoglycemic medication. Hypercholesterolemia was defined as a total cholesterol level of *≥*5.17 mmol/L, low-density lipoprotein cholesterol level of ≥3.38 mmol/L, or current use of cholesterol-lowering medication. Cigarette smoking was defined as present if the participant reported smoking at least 10 cigarettes per day during the past 5 years.

### Clinical Parameters

Stroke severity (National Institutes of Health Stroke Scale, NIHSS) was assessed at admission and functional outcome (modified Rankin Scale; mRS) [18] was analyzed 3 months after AIS onset. Magnetic resonance imaging (MRI), including diffusion-weighted imaging (DWI), was performed within 48 hours of admission using a 1.5-T MRI unit (Gyroscan; Philips Medical Systems, Eindhoven, the Netherlands). DWI infarct volume (in cm^3^) was calculated as the sum of the infarct area in each DWI slice × (slice thickness+interslice gap) [19].

### Blood Collection and Analysis

Baseline venous blood samples were collected at admission (D1; within 12 hours of symptom onset; median, 2.1 [interquartile range, 1.8–3.1] hours) prior to treatment from all enrolled patients (n = 105) and at the health screening from the selected controls (n = 34). Consecutive blood samples were acquired from the enrolled patients on day 2 (D2; 18–24 hours from baseline [D1]; n = 100) and day 3 of admission (D3; 36–48 hours from baseline; n = 48). All samples were centrifuged at 1,500×*g* for 15 min within 60 min of collection. The separated sera were stored at –76°C and used for YKL-40 and CRP testing. YKL-40 was measured using a commercially available enzyme-linked immunoassay kit (R&D Systems, Minneapolis, MN) according to the manufacturer’s protocol. Measurements were performed in duplicate, and the results were averaged. The intra- and inter-assay coefficients of variation were less than 8%. CRP was measured using a highly sensitive method with an automated immunonephelometric analyzer (Dade Behring, Deerfield, IL).

### Statistical Analyses

Group means were compared using the Student’s *t*-test (between 2 groups) or one way ANOVA test (more than 2 groups), and median values were compared with the Mann-Whitney *U*-test (between 2 groups) or Kruskal*-*Wallis test (more than 2 groups). Ratios were compared using the chi-square test. Median YKL-40 and CRP levels were compared using nonparametric tests because the concentrations were not normally distributed. If the median differences in YKL-40 or CRP levels were significant in a Kruskal*-*Wallis test, Conover’s post hoc tests were performed. Spearman correlation was used to assess the relationships between the clinical parameters (infarct volume, NIHSS, and mRS) and serum markers (YKL-40 and CRP).

Comparison of the diagnostic accuracies of YKL-40 and CRP for discriminating between AIS patients and the controls was performed by constructing receiver operating characteristic (ROC) curves. The statistical differences were analyzed using the Delong method. Multivariate logistic regression analysis was used to determine poor functional outcome.

Paired nonparametric Wilcoxon signed-rank tests were performed to compare serial changes in serum markers. Data were analyzed with MedCalc version 11.5 (MedCalc Software, Mariakerke, Belgium) and StatsDirect version 2.7.8 (StatsDirect Ltd, Cheshire, UK). Unless otherwise stated, a *P* value of less than 0.05 was considered statistically significant.

## Results

### Baseline Characteristics and YKL-40 and CRP Levels in AIS Patients and Controls

The demographic characteristics of the subjects are presented in Table 1. The age and sex distributions did not differ significantly between the control and AIS groups (*P*>0.05).

**Table 1:** Demographic Characteristics of AIS Patients and Controls.

		AIS Patients				*P*(Controlvs. AIS)
		Stroke subtype (TOAST)				
	Control	SVO	LAA	CE	All AIS	
	(n = 34)	(n = 42)	(n = 39)	(n = 24)	(n = 105)	
Age, y, mean (SD)	66 (11)	64 (9)	66 (13)	66 (10)	65 (11)	0.88
Male, n (%)	20 (59)	24 (57)	22 (56)	13 (54)	59 (56)	0.94
Diabetes mellitus, n (%)	4 (12)	11 (26)	8 (21)	8 (33)	27 (26)	0.14
Hypertension, n (%)	8 (24)	28 (67)	27 (69)	15 (63)	70 (67)	<0.001*
Hypercholesterolemia, n (%)	14 (41)	16 (38)	18 (46)	7 (29)	41 (39)	0.98
Smoking, n (%)	9 (26)	15 (36)	11 (28)	7 (29)	33 (31)	0.74
Previous stroke, n (%)	0 (0)	10 (24)	5 (13)	8 (33)	23 (22)	0.007*
NIHSS, median (IQR)	0 (0)	4.5 (3.0–6.0)	8.0 (4.0–14.0)	11.5 (5.0–16.0)	5.0 (4.0–11.0)	<0.001*
Infarct volume (cm^3^), median (IQR)	NT	0.7 (0.1–1.4)	7.2 (2.4–24.1)	6.0 (3.6–28.9)	2.7 (0.8–10.1)	NT
3-month mRS, median (IQR)	NT	1.0 (1.0–2.0)	1.0 (1.0–2.8)	3.5 (1.0–5.0)	1.0 (1.0–3.0)	NT

AIS, acute ischemic stroke; TOAST, Trial of Org 10172 in Acute Stroke Treatment criteria; SVO, small-vessel occlusion or lacunar; LAA, large artery atherosclerosis or atherothrombosis; CE, cardioembolic; NIHSS, National Institutes of Health Stroke Scale; IQR, interquartile range; NT, not tested; mRS, modified Rankin Scale.

* *P*<0.05.

**Figure 1:**
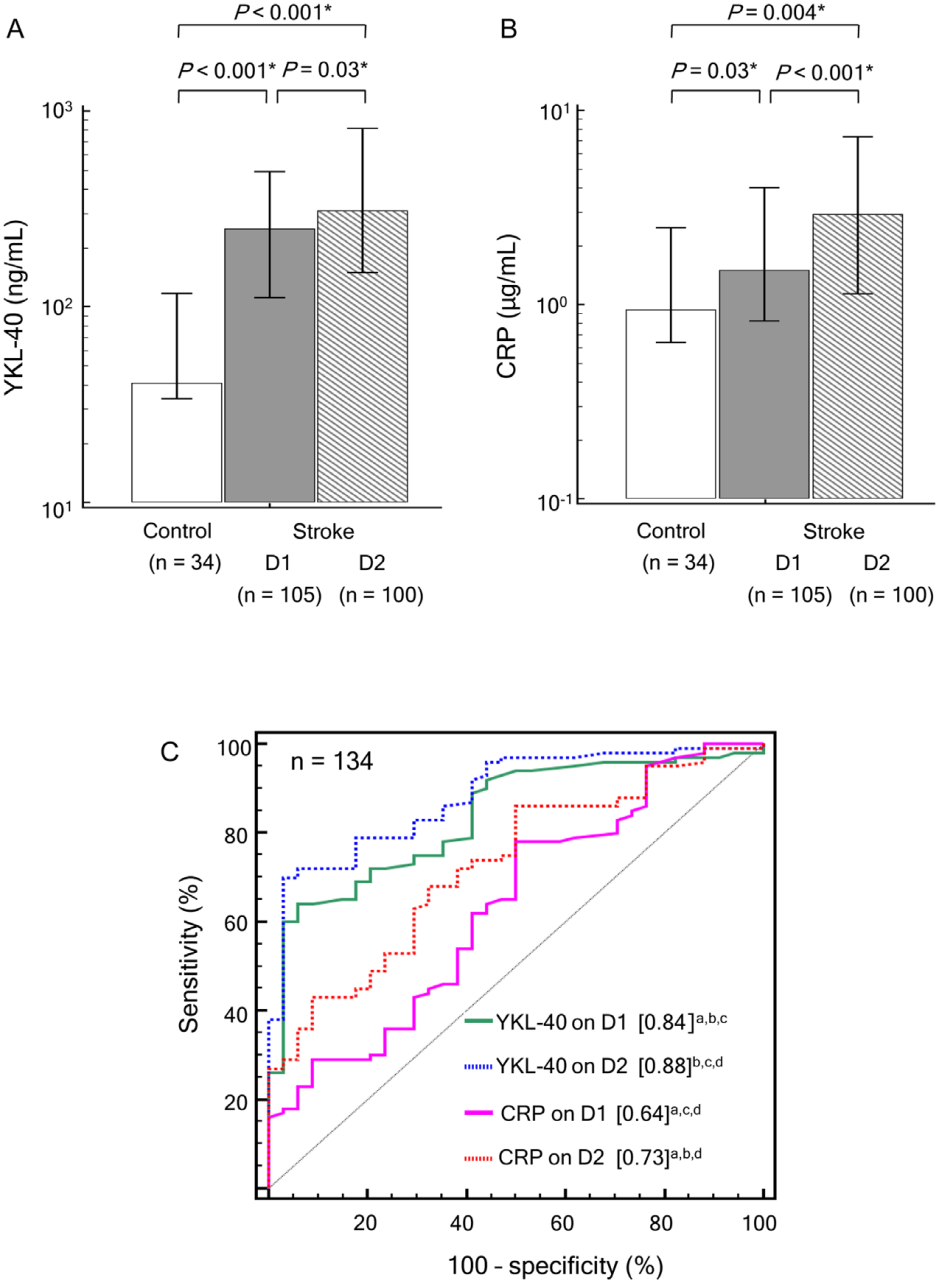
Levels of serum YKL-40 (A) and CRP (B) in acute ischemic stroke patients and controls. Each box indicates the median. Horizontal lines indicate the interquartile ranges. (**C**) Diagnostic accuracies of serum YKL-40 and CRP for discriminating acute ischemic stroke patients (n = 100; for statistical assessment of the differences between D1 and D2, 5 of 105 patients were excluded because they [n = 5] dropped out of the D2 test) from controls (n = 34) using receiver operating characteristic (ROC) curves. Numbers in square brackets indicate diagnostic accuracies (area under the ROC curves). D1, within 12 hours of symptom onset; D2, 18–24 hours from baseline (D1); CRP, C-reactive protein. *P<0.05. ^a^ *P*<0.05, vs. YKL-40 on D2. ^b^ *P*<0.05, vs. CRP on D1. ^c^ *P*<0.05, vs. CRP on D2. ^d^ *P*<0.05, vs. YKL-40 on D1.

The levels of serum YKL-40 on D1 and D2 were significantly higher in the AIS patients than in the controls (251 and 310 ng/mL, respectively, vs. 41 ng/mL; *P*<0.001; Fig. 1A). Serum YKL-40 levels of non-diabetic (D1 and D2>200 ng/mL, *P*<0.05) and diabetic (D1 and D2>200 ng/mL, *P*<0.05) AIS patients were significantly higher than those of non-diabetic (41 ng/mL) and diabetic (57 ng/mL) controls. This comparison result consistently held true for the presence or absence of other modifiable vascular risk factors (hypertension, hypercholesterolemia, or smoking).

The levels of serum CRP on both D1 and D2 were also significantly higher in AIS patients than in the controls (1.50 and 2.93 µg/mL, respectively, vs. 0.96 µg/mL; *P*<0.05; Fig. 1B). Both YKL-40 and CRP levels were higher on D2 than on D1.

### Diagnostic Accuracies of YKL-40 and CRP for Discriminating AIS Patients from Controls

We performed ROC curve analysis (Fig. 1C) and determined that the areas under the curves (AUCs) of YKL-40 showed good diagnostic accuracy. The AUCs of YKL-40 on D1 and D2 (0.84 and 0.88, respectively) were greater than those of CRP on D1 and D2 (0.64 and 0.73, respectively; *P*<0.05). For both markers, the AUC for D2 was greater than that for D1.

### YKL-40 and CRP Levels Depend on Stroke Subtype in Patients with Noncardiogenic AIS

**Figure 2:**
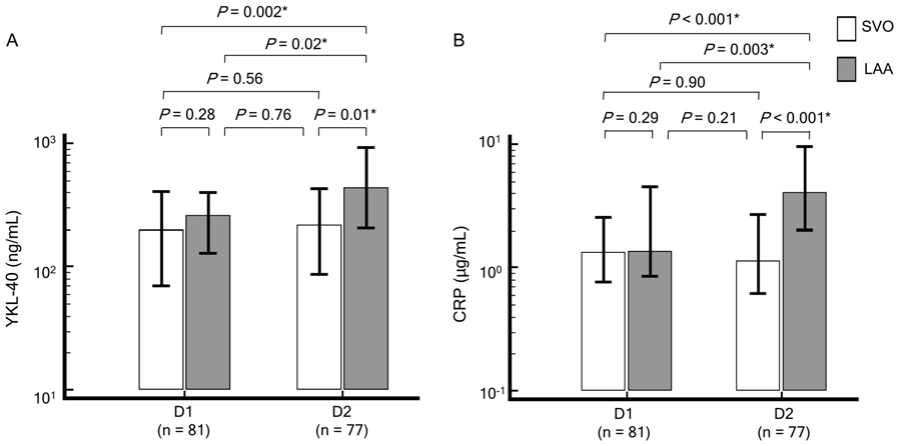
Levels of serum YKL-40 (A) and CRP (B) depend on stroke subtype (in noncardiogenic stroke). Each box indicates the median. Horizontal lines indicate the interquartile ranges. D1, within 12 hours of symptom onset; D2, 18–24 hours from baseline (D1); CRP, C-reactive protein; SVO, small-vessel occlusion or lacunar; LAA, large artery atherosclerosis or atherothrombosis. *P<0.05.

To determine the associations between AIS extent and YKL-40 and CRP levels in a homogenous etiologic (noncardiogenic) group, we analyzed YKL-40 and CRP levels in the context of 2 stroke subtypes (LAA and SVO) and found that the levels were dependent on AIS subtype and admission day (Fig. 2). YKL-40 levels on D1 (LAA vs. SVO, 263 vs. 201 ng/mL) tended to be higher in patients with LAA than in patients with SVO; however, this difference was not statistically significant (*P* = 0.28). D1 CRP levels (LAA vs. SVO, 1.37 vs. 1.36 µg/mL) did not differ between the subtypes (*P* = 0.29). On D2, both proteins were higher in LAA patients than in SVO patients (YKL-40∶444 vs. 218 ng/mL; CRP: 4.06 vs. 1.15 µg/mL, for LAA vs. SVO, respectively). In LAA patients, levels of both proteins were higher on D2 than on D1. In SVO patients, although the levels of serum YKL-40 were slightly higher on D2 than on D1, the difference was not statistically significant, and CRP levels did not differ by admission day.

### Correlation of YKL-40 and CRP Levels with Stroke Severity, Infarct Volume, and Functional Outcome

To evaluate the association between YKL-40 levels and the clinical characteristics of AIS, correlation analyses were performed (Table 2). On D1, the levels of YKL-40 (r = 0.26, *P*<0.001) but not CRP (0.13, *P* = 0.20) were correlated with 3-month mRS. In contrast, YKL-40 levels on D1 were not correlated with NIHSS or infarct volume. CRP levels on D1 were correlated with NIHSS but not with infarct volume. On D2, the levels of both proteins were correlated with all clinical characteristics examined, including NIHSS, infarct volume, and 3-month mRS. Additionally, both proteins correlated with each other on D1 and D2.

**Table 2:** Correlation of YKL-40 and CRP Levels with Stroke Severity, Infarct volume, and Functional Outcome.

	Spearman's rank	YKL-40	YKL-40	CRP	CRP	NIHSS	Infarct
	correlation	(D1)	(D2)	(D1)	(D2)		volume
YKL-40	r	0.90					
(D2)	*P*	<0.001*					
	n	134					
CRP	r	0.25	0.21				
(D1)	*P*	0.003*	0.02*				
	n	139	134				
CRP	r	0.38	0.46	0.74			
(D2)	*P*	<0.001*	<0.001*	<0.001*			
	n	134	134	134			
NIHSS	r	0.13	0.28	0.24	0.46		
	*P*	0.17	0.004*	0.01*	<0.001*		
	n	105	100	105	100		
Infarct	r	0.08	0.24	0.05	0.38	0.49	
volume	*P*	0.44	0.02*	0.62	<0.001*	<0.001*	
	n	105	100	105	100	105	
3 month	r	0.26	0.29	0.13	0.36	0.42	0.50
mRS	*P*	<0.001*	0.003*	0.20	<0.001*	<0.001*	<0.001*
	n	105	100	105	100	105	105

D1, within 12 hours of symptom onset; D2, 18–24 hours from baseline (D1); CRP, C-reactive protein; NIHSS, National.

Institutes of Health Stroke Scale; mRS, modified Rankin Scale.

* *P*<0.05.

### Role of YKL-40 as a Predictor of Functional Outcome

To evaluate the role of serum YKL-40 as an independent predictor of functional outcome, crude and multivariate-adjusted odds ratios (ORs) for poor outcome (3-month mRS 4–6) were determined (Table 3). The OR for poor functional outcome was greater in the highest tertile (5.73, *P* = 0.03) than in the lowest tertile of D2 YKL-40 in AIS patients after adjusting for age, sex, hypertension, diabetes mellitus, hypercholesterolemia, smoking, previous stroke and D2 CRP level. Although the adjusted ORs for poor functional outcome tended to be greater in the highest tertile of D1YKL-40 (1.89–2.10) than in the lowest one, the result was not statistically significant (*P*>0.05). The crude and adjusted (age and gender) ORs for poor functional outcome tended to be greater in the highest tertile of D2 CRP (3.02, *P* = 0.09 and 2.74, *P* = 0.13, respectively) than in the lowest one; however, this tendency did not reach statistical significance.

**Table 3:** Multivariate-Adjusted odds ratios for Poor Functional Outcome Depend on D2 Serum YKL-40.

YKL-40 (D2)				
Tertile	Crude or adjusted OR^a^	95% CI	*P* value	Adjusted factors^b^
Middle	2.22	0.51–9.76	0.29	None
Highest	5.46	1.37–21.7	0.02^c^	None
Middle	1.89	0.42–8.55	0.41	A
Highest	4.45	1.09–18.18	0.03^c^	A
Middle	2.13	0.46–9.76	0.33	B
Highest	5.61	1.25–25.21	0.03^c^	B
Middle	2.18	0.46–10.35	0.32	C
Highest	5.73	1.17–28.08	0.03^c^	C

D2, 18–24 hours from baseline; OR, odds ratio; CI, confidence interval.

a Reference OR (1.00) is the lowest tertile of YKL-40 for poor outcome (mRS 4–6).

b Adjusted factors: A = age and sex; B = A + hypertension, diabetes mellitus, hypercholesterolemia, and smoking; C = B+ previous stroke and D2 C-reactive protein level.

c *P*<0.05.

### Temporal Changes in YKL-40 and CRP Levels Following AIS

**Figure 3:**
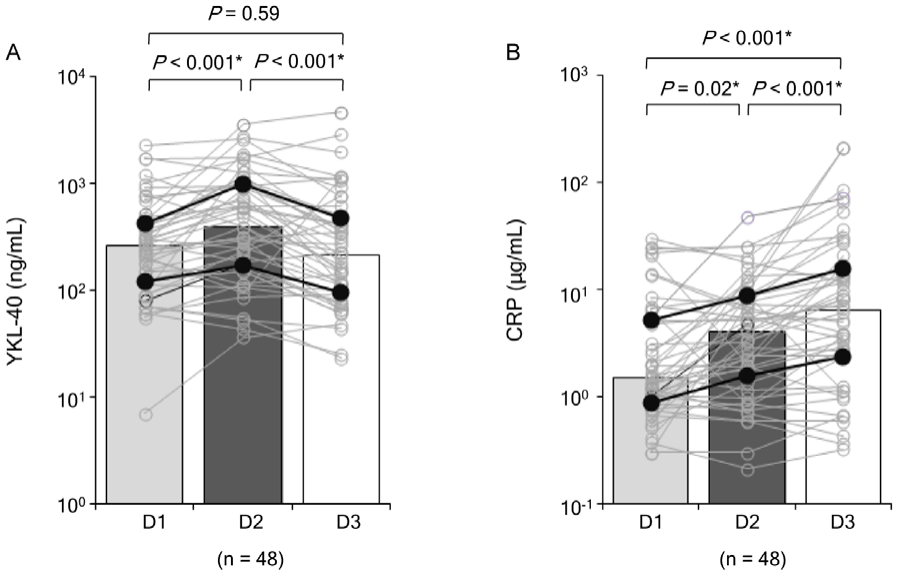
Temporal changes in YKL-40 (A) and CRP (B) levels following acute ischemic stroke. Open markers indicate serially analyzed protein levels in each patient. Each box indicates the median, and the closed markers indicate the interquartile ranges. D1, within 12 hours of symptom onset; D2, 18–24 hours from baseline (D1); D3, 36–48 hours from baseline; CRP, C-reactive protein. *P<0.05.

To further clarify the temporal dynamics of YKL-40 and CRP levels following AIS, both proteins were serially analyzed for the first 3 consecutive days of admission (D1, D2, and D3) in AIS patients (n = 48; Fig. 3). In most patients (58%, 28/48), YKL-40 peaked on D2, and the median values on D1, D2, and D3 (258, 391, and 214 ng/mL, respectively) verified that YKL-40 levels peaked on D2. We observed that in the majority of patients (67%, 32/48) CRP levels were highest on D3, and the median values of CRP on D1, D2, and D3 (1.50, 4.06, and 6.43 µg/mL, respectively) also showed that levels were highest on D3.

## Discussion

For the first time, our study demonstrated temporal changes in serum YKL-40 levels during the early phase of AIS and their role as a diagnostic and monitoring indicator of AIS.

Ischemic stroke is closely related to neuroinflammation, from pre-stroke atherogenesis to post-stoke brain injury [9], [10], [20]. Atherosclerotic plaque growth and rupture in cerebral vessels entail macrophage recruitment or release of immune mediators. Focal brain ischemia triggers not only local neuroinflammation induced by cell death but also systemic inflammation caused by disruption of the blood brain barrier [10], [20]. IL-1β and TNF-α, the major YKL-40 inducer cytokines, are synthesized from microglia and macrophages in penumbra after ischemic stroke [9], [10]. Based on these data, the observed elevation of circulatory YKL-40 levels in AIS patients compared to controls in the current study is a reasonable finding and consistent with data from a previous study that showed a remarkable elevation of YKL-40 in cerebrospinal fluid from stroke patients [1].

Notably, our study demonstrated that YKL-40 measurements are more useful for discriminating AIS patients from controls during the first 2 consecutive days of admission than are CRP measurements. This result suggests that YKL-40 more accurately reflects neuroinflammation or injury than does CRP, which is explained by the following postulation. Circulatory YKL-40 in AIS patient is mainly released from astrocytes in brain parenchyma in response to local neuroinflammation [1], [4], [5]. Therefore, circulatory YKL-40 levels rapidly, specifically, and directly, reflect neuroinflammation without the need for a secondary mediator, and are also capable of indicating the presence of a tiny infarction. In contrast, circulatory CRP is mainly released from hepatocytes in the liver in response to any systemic inflammation [6]. Thus, the CRP response may require a secondary mediator or a certain threshold for local neuroinflammation to switch to systemic inflammation (i.e., very slight neuroinflammation may resolve without causing systemic inflammation). Thus, CRP levels may relatively slow, nonspecific, and insensitive to microinfarctions, in contrast to YKL-40 levels.

Generally, the LAA type has greater atherosclerotic burden and infarction-associated injury than the SVO type [14], [17]. Therefore, we hypothesized that the LAA type is more closely associated with elevated YKL-40 levels than is the SVO type. The D2 results of our study fairly well reflected the expected characteristics of each subtype (i.e., LAA type presented higher levels of YKL-40 on D2 than SVO type and only the LAA type presented a significant elevation on D2 compared to D1). Although we could not determine whether chronic stable atherosclerotic lesion or acute injured lesion by infarction contributed more to the difference in serum YKL-40 levels between LAA and SVO, we postulate that the differences in YKL-40 levels on D2 between the 2 subtypes was more attributable to acute ischemic neuronal injury or peri-infarction inflammation than to chronic stable atherosclerotic lesion due to the rapid elevation in median YKL-40 levels on D2 compared to D1 only in LAA type AIS.

Despite the close association between YKL-40 and neuronal injury [1], [4], no previous study determined whether or how rapidly circulatory YKL-40 could reflect cerebral infarct volume. In our correlation analysis of protein levels and clinical parameters at admission, YKL-40 levels in AIS patients were not significantly correlated with infarct volume. In contrast, an association between CRP and cerebral infarct volume has been shown in several studies, but the findings are conflicting. Two studies showed a significant correlation [14], [21], while another 2 failed to find a relationship [22] or showed a dependency on follow-up time [15], which is in agreement with our findings. We hypothesize that the difference between our result and those of the former studies is primarily due to the timing of blood collection. The study that failed to show a correlation between CRP and infarct volume used blood samples that were collected within 24 hours of symptom onset [15], [22] which was similar to our admission blood collection protocol (within 12 hours of symptom onset). Conversely, the studies that showed a significant correlation included blood samples that were collected more than 24 hours after symptom onset [14], [21]. From the view point of the timing of blood collection, D1 YKL-40 levels were superior to D1 CRP levels, since a significant correlation was observed between D1 YKL-40 levels and functional outcome at the 3-month time point, while no significant correlation was observed for D1 CRP levels. In contrast, on D2, both proteins were useful as clinical status indicators for AIS and showed significant correlations with all 3 key clinical parameters. Furthermore, the D2 YKL-40 level was useful as an independent predictor of poor functional outcome after adjustment for age and vascular risk factors with adequate statistical significance. However, regardless of the correlation between D2 CRP and 3-month mRS and the tendency for higher ORs for poor outcome in the highest D2 CRP tertile, the D2 CRP level was not an independent predictor of poor functional outcome due to a lack of statistical significance. This may be because it was measured too soon after AIS onset to detect elevated CRP, or it could be due to an insufficient number of patients in this study [11], [12], [16]. In the serial analysis of each AIS patient, YKL-40 peaked earlier and declined more rapidly than CRP. Taken together, these data support the utility of YKL-40 as a prognostic serologic marker for the early phase of AIS and suggest that it may be an earlier indicator for halted of AIS progression than CRP.

Our study has some limitations. First, it does not allow for identification of the releasing source of circulating YKL-40. Various factors and medical conditions, including age, obesity, atherosclerosis, modifiable vascular risk factors, chronic neurological disorders (e.g., Alzheimer's disease, chronic infarction, and autoimmune neuronal disease) as well as acute infectious diseases may affect YKL-40 levels [1]–[4], [23]. Therefore, we could not exclude the possibility that the difference between the controls and AIS patients was caused by other YKL-40-elevating causes rather than AIS. However, considering the rapid elevation and subsequent decrease in YKL-40 within 3 days of AIS and the use of exclusion criteria, including acute infection and specified comorbidities, AIS seems to be the major contributor to the observed difference between AIS patients and controls, especially on D2. In addition, we assume that if the D2 YKL-40 elevation was more affected by secondary infection after AIS or other post-stroke complications than by AIS itself, YKL-40 levels would continue to increase on D3 instead of decline. Additionally, the significant differences in serum YKL-40 levels between AIS patients and controls were consistently observed regardless of the presence or absence of each modifiable vascular risk factor. Second, as a pilot study for YKL-40 levels immediately following AIS, our study has a limited sample size and subtype analysis. Although we did not observe a significant correlation between D1 YKL-40 levels and stroke severity or infarct volume, considering the tendency for higher D1 YKL-40 levels in patients with the LAA type of AIS a larger sample size would be desirable for further clarification of these relationships. Moreover, detailed subtype analysis, including other determined and undetermined causes, is required to elucidate that YKL-40 is generally an applicable monitoring indicator for all etiologic types of AIS.

Overall, our study identified dynamic alterations of serum YKL-40 levels following AIS and demonstrated that YKL-40 measurement is superior to that of CRP for discerning AIS patients from stroke-free elderly controls. In addition, we provided evidence that YKL-40 is an indicator of infarct volume, stroke severity, and functional outcome of AIS. The data presented here support the use of serum YKL-40 as an early surrogate marker for AIS extent and prognosis.
